# Physicochemical Composition, Antioxidant Status, Fatty Acid Profile, and Volatile Compounds of Milk and Fresh and Ripened Ewes’ Cheese from a Sustainable Part-Time Grazing System

**DOI:** 10.3390/foods10010080

**Published:** 2021-01-03

**Authors:** Rosario Gutiérrez-Peña, Carmen Avilés, Hortensia Galán-Soldevilla, Oliva Polvillo, Pilar Ruiz Pérez-Cacho, José Luis Guzmán, Alberto Horcada, Manuel Delgado-Pertíñez

**Affiliations:** 1 Departamento de Ciencias Agroforestales, Escuela Técnica Superior de Ingeniería Agronómica, Universidad de Sevilla, 41013 Sevilla, Spain; charo-84@hotmail.com (R.G.-P.); albertohi@us.es (A.H.); 2 Departamento de Bromatología y Tecnología de los Alimentos, Campus de Rabanales, Universidad de Córdoba, 14071 Córdoba, Spain; v92avrac@uco.es (C.A.); bt1gasoh@uco.es (H.G.-S.); pilar.ruiz@uco.es (P.R.P.-C.); 3 Servicio General de Investigación Agraria, Escuela Técnica Superior de Ingeniería Agronómica, Universidad de Sevilla, 41013 Sevilla, Spain; oppolo@us.es; 4 Departamento de Ciencias Agroforestales, Escuela Técnica Superior de Ingeniería, ‘Campus de Excelencia Internacional Agroalimentario, ceiA3’ Campus Universitario de la Rábida, Carretera de Huelva-Palos de la Frontera s/n., Universidad de Huelva, 21819 Huelva, Spain; guzman@uhu.es

**Keywords:** antioxidant capacity, dairy product quality, n-3 and n-6 fatty acids, retinol, Roja Mallorquina sheep, tocopherol, total phenolic compounds, volatile compounds

## Abstract

We conducted the first nutritional analysis of dairy products from the traditional Roja Mallorquina sheep breed. Samples of bulk raw milk were taken twice a month from December 2015 to March 2016 from sheep fed using a part-time grazing system, and fresh soft (FC, *n* = 8) and ripened (RC, *n* = 8) cheeses were made. The variability in vitamins, total phenolic compounds (TPC), total antioxidant capacity (TAC), and fatty acid (FA) content was influenced by the cheese-making process (differences between the cheese and the original milk) and by the type of cheese-making technology (mainly related to heating, the use of starter culture, and ripening). The most notable physicochemical characteristic of the cheeses was low fat content (24.1 and 29.6 g/100 g for FC and RC). Milk and RC were characterised by major concentrations of retinol (211.4 and 233.6 μg/100 g dry matter (DM), respectively) and TPC (18.7 and 54.6 μg/100 g DM, respectively), while FC was characterised by major concentrations of retinol (376.4 μg) and α-tocopherol (361.7 μg). The fat-soluble components of the FC generally exhibited better nutritional value for human health than those of the milk and RC, with a higher level of retinol and α-tocopherol; lower values for saturated FA, atherogenic, and thrombogenic indices; and higher levels of monounsaturated FA, polyunsaturated FA, n-3, and n-6. Acids, alcohols, and ketones comprised almost 95% of the volatile compounds detected. Acetoin and products of lactose and citrate metabolism played an important role in the development of the aromatic attributes of both kinds of cheese. This preliminary study can contribute to add value to these traditional products according to healthy nutritional criteria and supports the implementation of strategies to promote their commercialisation and obtain product labelling as “pasture-fed” or specific marks.

## 1. Introduction

The disappearance of many pastoral farming methods in Europe has revealed the importance of sustainable livestock management for environmental conservation [1]. In the Balearic Islands (Spain), traditional sheep systems based on the use of autochthonous breeds deserve to be highlighted. Many of these breeds, such as the Roja Mallorquina, are considered endangered according to the Official Catalogue of Spanish Livestock Breeds [2]. The Roja Mallorquina sheep is a meat breed raised under grazing systems. However, traditionally, some breeders have produced artisanal Mallorquin cheeses with a highly characteristic flavour attributed to the breed’s very fatty milk. Normally, two types of cheese are made: a fresh soft cheese (white in the beginning and slightly yellowish after ten or fifteen days) and a cheese ripened for approximately two months [3]. To our knowledge, no scientific data have been published so far concerning milk or fresh and ripened cheeses from the Roja Mallorquina.

Cheeses made from small ruminants’ milk are widely appreciated for their organoleptic properties. The composition of the lipids plays an essential role in the sensory traits of these products. However, dairy products have often been associated with many negative health effects due to their saturated fatty acid (SFA) content; this may lead to increased low-density lipoprotein cholesterol (LDL) levels and thus an increased risk of cardiovascular disease (CVD) [4]. Nevertheless, recent research has demonstrated the benefits of full-fat dairy consumption [5] and suggests that milk has a neutral effect on cardiovascular health, whereas fermented milk and cheese may be beneficial. Furthermore, foods containing natural antioxidants have grown in popularity, as antioxidants can neutralise harmful effects of free radicals both in humans and animals and because oxidation processes in milk can result in a deterioration of its nutritional quality [6,7]. However, few studies have investigated the milk and cheese characteristics of endangered sheep breeds.

Moreover, there is interest in assessing the effects of different feeding regimes on dairy quality, including the pathways that transfer nutritional components from milk to cheese and other dairy products. On the one hand, several feeding strategies, including grass feeding and pasture grazing, are known to confer specific organoleptic features on the dairy products while improving nutrition [8]. Moreover, in some European countries (including Spain) and mainly in cow dairy (most likely due to their large volume and economic importance), industry or marketing intermediaries fund farmers more for milk produced in grassland conditions [9,10,11]. Although the small ruminant dairy sector is more limited [10] and, in Spain, incentive payments for pasture-fed milk do not yet exist, labelling products as “pasture-fed” could improve the profitability of farms and industries. On the other hand, while numerous studies have been carried out on FA transfer, results vary: some authors have suggested that the FA profile of fresh or ripening cheese reflects that of the milk from which it has been made [12,13,14], while others [15,16,17] have suggested that alterations to the FA profile occur during ripening. However, few studies have specifically addressed the transfer of fat-soluble vitamins from milk to cheese [18], and no reports are available on phenolic compounds. Therefore, the aims of the present work were (i) to perform a nutritional analysis of the dairy products (milk, fresh and ripened cheeses) of the Roja Mallorquina sheep breed, including the determination of their physicochemical, antioxidant, fatty acid (FA), and volatile properties and (ii) to identify and explain changes in nutritional composition during the manufacturing procedure.

## 2. Materials and Methods

### 2.1. Study Area, Experimental Farm, and Feeding Management

The study was conducted on Mallorca (Balearic Islands) in the Mediterranean Sea in eastern Spain. A farm of indigenous Roja Mallorquina ewes was used in the present study. There are 55 farms included in the Breed registry of the Roja Mallorquina, with an average size of 73 adult animals per farm [2]. It is a rustic breed, and it is a good dam with good dairy conditions. The reproductive management of farms is discontinuous. Males are introduced into the female herd between May and November. Therefore, the lambing period is concentrated between September and April, coinciding with maximum forage production.

The management system is fundamentally based on grazing. Cultivated pastures, such as oats (*Avena sativa*), ryegrass (*Lolium* sp.), and barley (*Hordeum vulgare*), are used for animal feed in the form of green forage (direct grazing or cut grass), dry or ensiled forage, grains, and stubble. The natural grazing area is characterised by pastures with low arboreal stratum density of pine trees (mainly, *Pinus halepensis*) that allow the development of the herbaceous layer (most commonly *Dactylis glomerata*, *Brachypodium retusum,* and *Ampelodesmos mauritanica*) and the shrub layer (*Pistacia lentiscus*, *Rhamnus alaternus*, *Erica multiflora,* and various *Cistus* species) [19]. Natural grass surfaces are found to a lesser extent (most commonly *Dactylis glomerata*, *Hyparrhenia hirta,* and *Brachypodium retusum*) [19]. In winter and spring, the sheep graze on natural and cultivated pastures, while in summer and early autumn, they take advantage of stubble and graze on wooded areas. To meet the nutritional requirements of late pregnancy and lactation, ewes consume purchased grains (0.9 kg/ewe and day) and self-produced forage (from 0.3 to 1.1 kg/ewe and day, depending on pasture yield). Samples from all the feeds supplied to the ewes were collected, and their chemical composition was analysed (Table 1).

### 2.2. Milk Collection, Cheese Manufacture and Sampling

After weaning the lambs in November, the dams were milked with an automated parallel milking machine once a day for cheese manufacture from December 2015 to March 2016 (approximately from the middle to the end of lactation). The dams were included in the milking programme according to when their lambs were weaned, and they were removed once they were dried off, but the number of milking ewes remained stable over these months (an average of 150 animals). After homogenising the total yield of daily milk using a mechanical shaker, bulk raw milk samples were taken twice a month (*n* = 8, four replicate samples for each stage of lactation) for cheese-making. Five aliquots of whole milk from each sample were placed in 50 mL plastic bottles for chemical analyses. The sheepherder made cheese in two vats with the same bulk milk sample, one to make fresh soft cheese (FC, *n* = 8) and the other to made ripened cheese (RC, *n* = 8). The cheeses were made following traditional manufacturing conditions. Overall, 16 cheese-making processes were completed. The FC was made with pasteurised milk and without adding starter culture. Milk was pasteurised at 63 °C for 30 min and cooled to the ripening temperature (34 °C). Calf rennet commonly used by farmers (>60% chymosin and <40% pepsin) was added in the amount of 1 mL per 3 L of milk, following the manufacturer’s instructions, to obtain clotting within 45 min. After coagulation, all the curds were cut to obtain grains the size of millet. Salt (NaCl) was added to this mixture in a proportion of 1%, w/v. The curd was stirred manually for approximately 20 min, and then the whey was drained. The curd was packed into cylindrical moulds (500 g), and the cheeses were transferred to an airing chamber at a temperature of 3–4 °C without humidity control. The cheeses were sold the next day with a shelf life of 8 days in refrigeration.

The RC was made with starter culture added to unpasteurised milk. After heating to 34 °C, the ferment was added in the proportion of 6 g per 100 L of milk (Mesophilic Starter Series MA 4001, Dupont Nutrition and Biosciences Ibérica, Valencia, Spain). After 30 min of ripening, calf rennet was added in the same proportion to obtain clotting in 45 min. After coagulation, the curd was cut with a lyre of parallel wires to obtain grains the size of hazelnuts. The curd was stirred semi-mechanically for 30–40 min, and then the whey was drained. The curd was packed into cylindrical moulds (500 g or 1 kg) and pressed in a hydraulic press (2.4 kPa) for 1 h. Then, they were salted by brining in NaCl solution (6 °C, 16° Bau, and pH 5.15–5.20) for 8 h. Afterwards, the cheeses were transferred to an airing chamber for 72 h at 12 °C and 80% relative humidity. Finally, the cheeses were transferred to a maturation chamber for 60–70 days of storage at 13 °C and 85–90% relative humidity.

The milk samples and cheeses were transported in a portable cooler with ice. Once in the laboratory, the cheeses were cut into sections (approximately 200 g each) and vacuum-packed. All milk and cheese samples were wrapped in aluminium sheets to preserve them from light and frozen at −20 °C until analysis, except the samples for the analysis of vitamins, which were frozen at −80 °C.

### 2.3. Chemical Analyses

Before analysis, the feed samples were dried and ground using a Wiley mill with a 1 mm screen. AOAC methods [20] were used to determine dry matter (DM, method 934.01), crude protein (method 984.13), crude fibre (method 978.10), and ash (method 942.05) content.

The milk’s basic chemical composition (DM, fat, protein, and lactose) was analysed with a Milkoskan FT (Fourier transform infrared analysis) in a CombiFoss 5000 (Foss Electric, Hillerød, Denmark) calibrated against known standards and subjected to quality controls and inter-comparative trials [21]. In cheese, the fat [22], DM [23], ash, and protein [24] contents were measured. For the determination of nitrogen content, the Kjeldahl method was used, and the total nitrogen content was multiplied by 6.38 to determine total protein. The sodium chloride content was analysed using a back titration with potassium thiocyanate to determine the concentration of chloride ions in the solution (the Volhard method) [23]. The pH was measured with a pH meter (pHmetro HANNA FHT-803) with a pH electrode, according to de la Haba et al. [25].

The procedures described by Guzmán et al. [26] were used to measure FA composition. Fat extraction from 0.1 g of freeze-dried milk or cheese and the direct methylation of FAs were performed in a single-step procedure based on the method by Sukhija and Palmquist [27] and revised by Juárez et al. [28]. Separation and quantification of FA methyl esters (FAMEs) were carried out using a gas chromatograph (Agilent 6890N Network GS System, Agilent, Santa Clara, CA, USA) equipped with a flame ionisation detector (FID) and automatic sample injector HP 7683, and fitted with an HP-88 J&W fused silica capillary column (100 m, 0.25 mm i.d., 0.2 µm film thickness; Agilent Technologies Spain, S.L., Madrid, Spain). Nonanoic acid methyl ester (C9:0 ME) was used as an internal standard (Sigma Aldrich Co., Madrid, Spain). Individual FAs were identified by comparing their retention times with those of the authenticated standard FA mix Supelco 37 (Sigma). The CLA (conjugated linoleic acid) isomers (*cis*-9, *trans*-11 and *trans*-10, *cis*-12) were identified by comparing retention times with those of another authenticated standard (Matreya, LLC, Pleasant Gap, USA).

Fat-soluble vitamin (A and E) analyses of 1.5–2 mL of milk or 2 g of cheese from each relevant sample were based on the methods by Herrero-Barbudo et al. [29] and Chauveau-Duriot et al. [30] and modified by Gutiérrez-Peña et al. [31]. Chromatographic analysis was carried out on an Acquity UPLC, with a fluorimetric detector and isocratic pump, PDA and 150 × 2.1 mm Acquity UPLC HSS T3 1.8 µm column (Waters, Saint-Quentin-en-Yvelines, France). Tocopherols and retinol were positively identified by comparing their retention times with those of high-purity standards of the measured substances (all-trans-retinol, α-tocopherol, β-tocopherol, and γ-tocopherol; Sigma Aldrich Co., Madrid, Spain). Other standards of high purity (retinyl acetate, retinyl palmitate, and tocopheryl acetate; Sigma Aldrich Co., Madrid, Spain) were used as internal standards. Since vitamins A and E are fat-soluble, it was necessary to normalise their concentrations against fat in order to determine how milk composition and the cheese-making process contributed to their variation among cheese samples [18].

Total antioxidant capacity (TAC) in milk was analysed by the ABTS method (2,2′-azino-bis (3-ethylbenzothiazoline-6-sulphonic acid)) of Pellegrini et al. [32], modified by Delgado-Pertíñez et al. [33]. Before any measurements were taken for milk, samples were sonicated for 10 min and diluted 10–20 times. Cheese samples for analysis were prepared according to the procedure of Revilla et al. [34] by diluting 2.5 mg of ground cheese in 10 mL of water. After stirring in a water bath at 40 °C, the mixture was centrifuged at 20 °C for 30 min (3000× *g*). The supernatant was recovered and brought up to a final volume of 10 mL, and a suitable amount of sample (20 μL) was used to measure TAC as in milk. The water-soluble vitamin E analogue Trolox was used as a standard, and TAC was expressed as mmol Trolox equivalents.

Total phenolic compounds (TPC) in milk were quantified according to the procedure described by Vázquez et al. [35] using the Folin–Ciocalteu method as modified by Guzmán et al. [26]. Cheese samples for analysis were prepared by diluting 10 g of ground cheese in 10 mL of methanol, and after vortexing for 40 min, the mixture was centrifuged at 5 °C for 10 min (3000× *g*). The supernatant was recovered, and then the same procedure as for milk was followed. Standard solutions of gallic acid (GA) were used to express the phenolic compounds as g of GA equivalents.

Following Guzmán et al. [36], 5 g of cheese samples were processed in order to extract their volatile compounds with solid-phase microextraction (SPME). A 1 cm long × 110 µm diameter divinylbenzene/carboxen/polydimethylsiloxane fibre (DVB-CAR-PDMS; Supelco, Bellefonte, PA, USA) was fixed in the headspace of the vial. Volatile compound analysis by gas chromatography-mass spectrometry (GC-MS) was also performed following Guzmán et al. [36]. The volatile compounds were tentatively identified by comparing their retention index (obtained using a series of n-alkanes in diethyl ether analysed under the same conditions) to those previously described in the literature and/or comparing their mass spectra with those in the National Institute of Standards and Technology library (NIST; Gaithersburg, MD, USA).

All chemical determinations were made in duplicate.

### 2.4. Data Processing and Statistical Analysis

The data on the nutritional composition of the three dairy products collected from each of the eight cheese-making sessions (four in each phase of lactation) were analysed using IBM SPSS Statistics for Windows (version 25.0; IBM Corp., Armonk, NY, USA). The normality of variables was assessed using descriptive statistics for asymmetry and kurtosis and the homogeneity of the variances was estimated with Levene’s test. To meet the objectives of this study, we used contrasts to investigate the following phenomena. (i) The first was the effects of manufacturing procedures between original milk and cheese (M vs. FC; M vs. RC) on fat-soluble vitamins, TPC, TAC, and FA composition (contents, proportions, categories, and indices). The data were analysed with the repeated measures procedure, and the model included fixed within-subjects factors for the product and lactation stage (repeated measures) as well as the interactions between these factors; the cheese-making session was the replicate. (ii) The second was differences among the fresh and ripened cheeses (FC vs. RC) on fat-soluble vitamins, TPC, TAC, and FA composition. The data were analysed with the repeated measures procedure, and the model included a fixed between-subjects factor for product and a fixed within-subject factor for lactation stage (repeated measures), as well as the interactions between these factors; cheese-making session was the replicate.

Only the product effect means are presented for both types of contrast, as the lactation stage effect and the interaction between product and lactation stage effects were not significant for most of the parameters analysed (*p* > 0.05). Furthermore, Pearson correlation coefficients were calculated for some of the variables used. Statistically significant differences and trends were defined as *p* ≤ 0.05 and 0.05 < *p* ≤ 0.10, respectively.

For the volatile compound content, a multivariate analysis was performed with the principal component analysis (PCA) command of the XLSTAT software (Addinsoft Inc., New York, NY, USA). The results were transformed by an orthogonal rotation (Varimax) with Kaiser normalisation (three components extracted).

## 3. Results

### 3.1. Physicochemical Composition and Antioxidant Status

Table 2 presents the means for the physicochemical parameters of fresh and ripened Roja Mallorquina ewes’ cheese and the corresponding original milk. The DM, fat, and protein contents of the milk ranges were 18.3–20.7, 6.58–8.72, and 6.08–7.31 g/100 g milk, respectively; the contents of FC ranges were 46.1–48.9, 22.2–25.0, and 11.9–17.1 g/100 g cheese, respectively; and the RC contents ranges were 62.2–65.5, 27.0–33.0, and 22.8–28.0 g/100 g cheese, respectively. The fat/DM ranges were 48.2–52.5 and from 41.2–52.5 g/100 g DM for FC and RC, respectively. Finally, the ash (g/100 g cheese), NaCl (g/100 g cheese), and pH ranges were 1.57–1.92, 0.88–1.54, and 6.43–6.65 for FC and from 3.04–4.14, 0.84–1.38, and 5.17–5.39 for RC.

Table 3 shows the fat-soluble vitamins, TPC, and TAC of the dairy products. The retinol and α-tocopherol contents of milk ranges were 113.4–281.2 and 53.1–156.8 μg/100 g DM, respectively; the contents of FC ranges were 218.9–520.7 and 208.5–549.3 μg/100 g DM, respectively; and the RC contents ranges were 180.0–310.1 and 11.7–49.9 μg/100 g DM, respectively. The TPC content of milk, FC, and RC ranges were 16.8–20.9, 4.0–8.0, and 34.8–69.8 mg GA (gallic acid) equivalents/100 g DM, respectively. The TAC content for milk, FC, and RC ranges were 7.4–50.4, 61.8–139.1, and 56.7–138.9 μmol Trolox equivalents/g DM, respectively. All these components were affected by cheese manufacturing. The fat-soluble vitamin contents (expressed as g DM and normalised against fat) were significantly higher in FC (376.4 and 361.7 μg/100 g DM, for retinol and α-tocopherol, respectively) than in milk (211.4, *p* < 0.05; 84.8, *p* < 0.01) and RC (233.6, *p* < 0.01; 32.6, *p* < 0.001). In this respect, an average of 49% of the retinol and 281% of the α-tocopherol originally present in milk fat were added during the cheese-making process. Furthermore, the retinol content in milk did not differ significantly from the RC (*p* > 0.05), but the content of α-tocopherol in milk was significantly higher than in RC (*p* < 0.05; 57% lost during the cheese-making process on average). The TPC content was significantly higher in RC (54.54 mg GA equivalents/100 g DM) than in milk (18.7 mg GA equivalents, *p* < 0.01; 193% average increase over milk content during the cheese-making process) or FC (6.16 mg GA equivalents, *p* < 0.001). Moreover, the TPC content in milk was significantly higher than in FC (*p* < 0.001; 67% on average lost during the cheese-making process). The TAC was significantly higher in cheeses (*p* < 0.05) than in milk (22.4, 100.0 and 102.5 μmol Trolox equivalents/g DM, for milk, FC, and RC, respectively).

### 3.2. Fatty Acid Composition

Table 4; Table 5 show the FA composition in mg/g DM and in relative percentages, respectively. Saturated FAs were predominant, constituting 73.3% of FAs in milk, 74.9% in RC, and 71.1% in FC (Table 5). Monounsaturated and polyunsaturated FAs reached a value of 20.9 and 5.8% for milk, 19.2 and 5.9% for RC, and 22.2 and 6.7% for FC, respectively. The major FAs were C16:0, C18:1 n-9 cis, C18:0, and C14:0 (with percentages higher than 9.5% of total FAs). The FA composition was affected by cheese manufacturing, especially in the case of milk compared with FC and in the case of FC compared with RC.

In terms of amount, FC showed higher content in the large majority of both individual FAs and FA groups compared with the original milk (Table 4). Regarding the health indices, a higher polyunsaturated FAs (PUFA)/SFA ratio was observed in FC (*p* < 0.05, Table 4). In terms of relative percentages, the differences were observed especially in the groups of the FAs, although the individual FA C12:0 and C18:1 n-9 cis also presented significant differences (*p* < 0.05) (Table 5). FC had a lower percentage of SFA (*p* < 0.05); higher percentages of monounsaturated FAs (MUFA), PUFA, and n6 (*p* < 0.05); and a tendency toward a higher percentage of n-3 (*p* < 0.10) compared to milk (Table 5).

RC had far fewer differences in FA composition compared to the original milk than FC. In terms of amount, the RC showed a higher content of total (*p* < 0.05) and individual (*p* < 0.01) short-chain FA (SCFA), C12:0 (*p* < 0.05), and CLA *cis*-9, *trans*-11 isomer (*p* < 0.05) (Table 4). Regarding the health indices, a higher atherogenic index (AI) and a lower MUFA/SFA ratio and health-promoting index (HPI) were observed in RC (*p* < 0.05, Table 4). Similarly, the percentages of FAs were affected by the product (Table 5); the RC showed higher values of individual SCFA (*p* < 0.01), C12:0 (*p* < 0.01), C14:0 (*p* < 0.01), and CLA *cis*-9, *trans*-11 isomer (*p* < 0.05) but significantly lower percentages of C18:0 (*p* < 0.001) and total n-3.

Comparing both types of cheese, the FC showed higher content in most FAs than the RC with the exception of short and medium-chain C4-C14 FAs and some other minority FAs that were not affected by the product (Table 4). Regarding the health indices, higher MUFA/SFA, PUFA/SFA, and HPI (*p* < 0.001) and a lower AI (*p* < 0.001) and thrombogenic index (TI, *p* < 0.01) were observed in FC. Similarly, except for C16:0 and CLA *cis*-9, *trans*-11 isomer, the FA percentages were affected by the product (Table 5). FC had lower percentages of total SCFA (*p* < 0.01), C12:0 (*p* < 0.001), C14:0 (*p* < 0.001) and total SFA (*p* < 0.001) and higher percentages for the rest of the individual FAs and FA groups.

### 3.3. Volatile Compounds

A total of 81 volatile compounds were detected: 17 acids, 17 alcohols, 16 ketones, 7 aromatic compounds, 8 esters, 5 aliphatic hydrocarbons, 2 sulphur compounds, 1 terpene, 3 aldehydes, and 5 lactones. Acids, alcohols, and ketones comprised almost 95% of the volatile compounds in both kinds of cheeses (FC and RC). However, acids were the most abundant family (39.7%) followed by alcohols (29.7%) in FC, while alcohols were the predominant compounds in RC (48.0%) followed by ketones (33.2%). Although both kinds of cheeses showed the same number of aromatic hydrocarbons, their concentration was higher in FC (3.0%) than in RC (1.2%). FC exhibited a greater quantity and variety of ester compounds and aliphatic hydrocarbons than RC (2.1% vs. 0.6%). Just one sulphur compound was detected in FC, but its concentration was much higher than the two detected in RC combined (0.15% vs. 0.06%). Only aldehydes and lactones were more abundant and varied in RC (0.21% and 0.31% respectively) than in FC (0.09% and 0.03%).

Acetic and 3-methylbutanoic acids were the most abundant short-chain FAs detected in FC, while hexanoic and octanoic acids predominated in RC. Ethanol was one of the predominant alcohols in both FC and RC samples. Moreover, 3-methyl-1-butan-ol and 2-butanol were present in high concentrations in FC and RC, respectively; the latter was the most concentrated volatile compound detected in RC. Butan-2-one-3-hydroxy and butan-2-one were the most abundant ketones identified in FC and RC, respectively. The former was also the predominant volatile organic compound in FC, while the latter was not present in this type of cheese. Benzene-ethanol, toluene, and *p*-xylene were the most abundant aromatic compounds that were detected in both kinds of cheese. Regarding ester compounds, ethyl acetate was the most concentrated in FC, while ethyl hexanoate had the highest concentration in RC. Lactones such as δ-octalactone and δ-decalactone were detected in both kinds of cheese, albeit in low quantities. Decanal was the only aldehyde identified in FC, while 4-heptanal was the most abundant in RC. Other compounds belonging to various minority families in this kind of product such as pentadecane, 2-ethylthio-ethanol, and farnesol were detected in both kinds of cheese.

**Figure 1:**
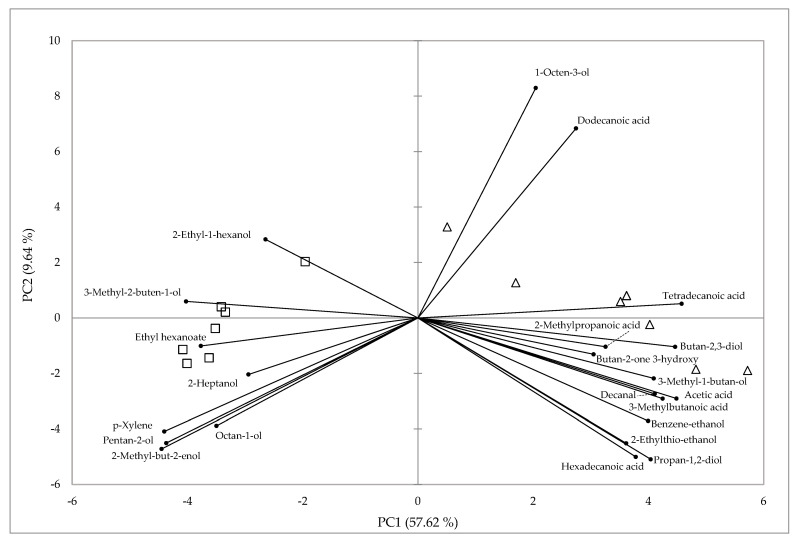
Principal component analysis plot representing the differentiation of Roja Mallorquina ewes’ cheeses based on the main volatile compounds. △, fresh soft cheese; □, ripened cheese.

The PCA was performed using the most discriminating volatile organic compounds present in both kinds of cheeses. The first two components (PC1 and PC2) accounted for 67.3% of the total variance, with PC1 and PC2 contributing 57.62% and 9.64%, respectively (Figure 1). FC and RC were clearly separated by PC1. Acids, alcohols, one aldehyde, and one ketone contributed positively to PC1, while alcohols, one ester, and one aromatic compound contributed negatively to PC1.

## 4. Discussion

### 4.1. Physicochemical Composition and Antioxidant Status

It is well known that several interacting factors reflect the characteristics of the original milk (breed and feeding) and its treatment (raw or pasteurised and enriched with selected starter cultures) as well as the conditions of cheese-making and ageing [17]. The breed and the ripening time are the most influential factors on the physicochemical characteristics of cheeses [25].

In general, the DM, fat, protein, and lactose contents of the milk used for the manufacture of Roja Mallorquina cheese were lower than in Guirra and Manchega breed milk [39] and higher than Assaf breed milk [40]. The main compositional characteristic of Roja Mallorquina ewes’ cheese was its low fat content. These results contrast with other studies of Spanish sheep milk cheeses such as Idiazabal [41], Roncal [42], Manchego [43], and Los Pedroches [44], which found higher fat/DM content than our study. However, our results fall within the range of minimum fat/DM allowed by the European Commission’s PDO regulations for Spanish sheep milk cheeses. With respect to pH, our values for ripened cheese agree with other studies [40,42,43,44].

Studies of fat-soluble vitamins in Spanish sheep dairy products are scarce in the scientific literature; to our knowledge, no research is available on FC. In addition, research on TPC is lacking. Regarding the vitamins, the most abundant compound in milk and RC samples was retinol, while the FC showed similar contents of retinol and α-tocopherol. Our results differ from those of Valdivielso et al. [13,45], who evaluated the fat-soluble vitamins of Spanish sheep cheeses manufactured with milk from flocks fed in grazing systems. These authors found a higher content of both vitamins, as well as the inverse pattern of α-tocopherol concentrations compared to our work (higher content in ripened cheeses than in the original milk). The vitamin contents may vary because the retinol and α-tocopherol contents of milk and cheese depend on many factors, such as the amount of herbage intake, the botanical and chemical composition of the pasture, the pasture’s vegetative stage, and the concentrate feed supply in animals’ diets [13,31,45].

The effect of the cheese-making process on the original milk characteristics depends on the manufacturing parameters, which thereby influence cheese features [18]. Lucas et al. [18] found that, on average, 34% of the vitamin A and 67% of the vitamin E in milk fat were lost during the cheese-making process. To a limited extent, these variations were explained by the milk fat composition, but the cheese-making process had a more substantial effect. In the present work, only the loss of α-tocopherol in the RC with respect to the original milk (over 57%) agree with these results. According to Lucas et al. [18], the loss of these micronutrients during the manufacturing process may result from their oxidative degradation by atmospheric oxygen and light as well as partial loss into the whey. In our study, the retention of these fat-soluble compounds, in particular retinol, during the cheese-making process (Table 3), could reflect the following possibilities: (a) the vitamin A of milk decreased rapidly during the first hours of light exposure, but it did not decrease further afterwards [46]; (b) casein is able to fix large quantities of retinol and α-tocopherol, and retinol that is bound to casein better resists degradation [47]; and (c) the salting process mediates the physicochemical conditions that alter the macromolecular structures of both protein and lipid molecules, and these modifications are also related to the retention of compounds in the cheese [48,49]. The increase of some fat-soluble compounds in FC, such as α-tocopherol (Table 3), may appear surprising. However, since cheese is a condensed product of milk, substances present in low concentrations in milk are more likely to be detected and even have a higher concentration in cheese [13,50]. This could reflect losses of milk proteins, lactose, fat, and minerals into whey during curd stirring and draining [51]. Finally, the type of cheese-making technology (primarily implying differences in heating, the use of starter culture, and ripening) has an important effect on the cheese composition for fat-soluble vitamins, with a higher level of retention of retinol and α-tocopherol in FC. However, compositional variability was not significantly influenced by cheese-making technology in the study by Lucas et al. [18]. Therefore, our results are not easily explained but could reflect the different processing conditions, since these affect the loss of potential cheese constituents at any stage after milking [51]. Consequently, more research is needed to elucidate the relationship between the manufacturing parameters and the fat-soluble components of cheese.

TPC variability was influenced by the cheese-making process (differences between the cheese and the original milk) and by the type of cheese-making technology (differences between cheeses). The results of this study show higher TPC concentrations in unpasteurised products (milk and RC, Table 3), which is in agreement with the results obtained by Chávez-Servín et al. [52] for goat milk, whey, and cheese. Phenolic degradation is one of the adverse effects of thermal pasteurisation that can reduce TPC concentrations [53,54]. The higher content of TPC in the RC could also be a consequence of the higher concentration of these compounds during the cheese-making process.

The TAC variability between the samples was mainly influenced by the cheese-making process, which is in agreement with the results obtained by Lucas et al. [18]. However, in this work, and unlike our results, the TAC (using the ferric reducing/antioxidant power, FRAP) of the milk was higher than the TAC of the cheese. The antioxidant capacity of natural antioxidants in dairy products is mainly due to phosphate, vitamins A and E, carotenoids, zinc, selenium, enzyme systems, oligosaccharides, and peptides [7]. They also may contain phenolic compounds [26,52,55], which have a direct effect on antioxidant activity in milk [55]. None of the antioxidant compounds analysed in this study were found to be correlated with the TAC either in milk or cheese, according to the ABTS method (data not shown). This may be because this method monitors the antioxidant capacity of both whey and total milk and is more sensitive to caseins and other low-molecular-weight compounds [56,57]. The high concentration of these proteins as a result of the loss of whey proteins and other water-soluble compounds during the cheese-making process could explain the higher TAC in cheeses than in milk.

### 4.2. Fatty Acid Composition

Studies involving the FA composition of Spanish sheep dairy products are limited to milk and RC, but to our knowledge, no research is available in FC. The results of FA composition in the present study are consistent with previous studies [13,45,57,58] that evaluated the FA profile of Spanish sheep cheeses manufactured in grazing systems. Milk FAs is influenced by animal feeding, and most studies on sheep grazing, as those previously mentioned, have reported that fresh pasture intake lowers the SFA content of fat, whereas that of some unsaturated FAs (UFA), such as vaccenic acid (VA), rumenic acid (RA), and n-3 FAs (ALA, α-linolenic acid; DHA, docosahexaenoic acid; EPA, eicosapentaenoic acid), increases [13,45,57,58]. With regard to the transfer of AG from milk to cheese, previous research has shown that for short (30 or fewer days) [14] or long periods of ripening (60–90 days or more) [12,13], the cheese’s FA profile was similar to that of the original milk. However, in agreement with the results of the present study, the FA profile of the long ripened cheeses differed from that of the original milk [15,16,17]. In terms of the amount of FA by g of DM, the content of FAs was higher in cheese than in milk, especially in FC (Table 4), which is in line with the results of Valdivielso et al. [13] for Spanish RC. The authors explained that the higher concentration of FAs in cheese that maintain the same FA profile as the milk could be due to losses of main constituents of milk during the cheese-making process. This explanation may be at least partially responsible for our results, but it is clear that the different FA profile between the milk and its derived cheese must be attributed to other causes. Although comparison of these results with those of earlier studies is difficult, given the different types of cheese reported in the literature; nevertheless, it is interesting to mention the following observations.

Bergamaschi and Bittante [59] evaluated the FA profile of different dairy products from cows grazing in highland pastures, and their comparative results between fresh cheese and cheese ripened for six months are in line with those of the present study (e.g., FC had lower SFA, and AI and TI indices; higher percentages of MUFA, PUFA, n-3, and n-6). These differences, which are also detected between the FC and the original milk, could be related at least partially to the relationship between the FAs and whey proteins, as well as the effect of the processing temperature of milk during the FC production process. Indeed, and according to previous works [59,60], the link between long-chain FAs and the major protein fractions in ricotta (β-lactoglobulins) should protect the FAs against isomerisation and oxidation reactions during the high processing temperature of ricotta production. In addition, the variation of FA during manufacture and ripening could be attributed to the lipase activity present, the rennet used, and the activities of microorganisms [15,59]. The activity of these lipases is especially related to the release of free FA, which has a significant impact on the development of the characteristic flavour of cheeses. In the present study, the RC samples analysed were characterised by higher concentrations of short- and intermediate-chain FAs (C4:0–C12:0, see Table 3). Generally, these FAs have a significant impact on the development of the characteristic flavour of cheese [61] because of their low perception threshold [62]. In terms of human health, the most favourable MUFA/SFA and PUFA/SFA ratios and AI, HPI, and TI indices were observed in milk and FC, suggesting that these products may be less harmful to human health than RC [37,38,63]. However, in all the products, the n-6/n-3 ratio remained within the recommended value to prevent CVD (less than four) [64] and was similar to previous studies of sheep and goats in comparable semi-extensive systems [13,31,33,58].

Ruminant products represent the primary dietary source of RA (CLA *cis*-9, *trans*-11), which are credited with health benefits [65]. Most geometrical and positional isomers of C18:1 and C18:2, such as CLA isomers, found in milk originate from microbial hydrogenation in the rumen and subsequent enzymatic desaturation of hydrogenated intermediates in the mammary gland [66], mainly from ALA and linoleic acid (LA). Several studies have highlighted the potential of pasture grazing for enhancing the CLA proportion in milk. Thus, Valdivielso et al. [45] showed that mountain sheep milk and cheese FA profile had healthy CLA isomers (mainly RA, ≈2% of the total FAs), which were derived essentially from the intake of LA and ALA: the prevalent FAs in pasture. Furthermore, in other studies by the same authors [13,57,58], the CLA content in both milk and cheese increased progressively from indoor feeding to grazing, particularly when sheep were under mountain or part-time valley grazing. In our study, pasture intake and FA composition were not measured, but the extensive feeding regime, similar to the part-time grazing of the aforementioned studies, may be responsible for the CLA results in milk. Interestingly, cheese manufacture increased the content of RA, but the percentage of this CLA isomer was only significantly higher in the ripened cheese (Table 4; Table 5). Enhanced CLA formation at this stage could be attributed to (a) the enzymatic isomerisation of LA to CLA [67]; indeed, we observed a relationship between the percentage of LA and RA (r = −0.64, *p* < 0.05) and (b) the migration of hydrogen on linoleic acid allyl radicals to form conjugated dienyl radicals, which react with hydrogen atoms from proteins to form CLA [67,68]. This process becomes more applicable as ageing progresses, as a consequence of concurrent enzymatic hydrolysis of proteins to low-MWF (molecular-weight fractions), which are better hydrogen donors than high-MWF [15,69]. In the case of FC (made with pasteurized milk), CLA production could be affected by temperature, although the influence of this factor during the manufacture of cheeses and other dairy products is controversial [70,71]. The authors have shown that UFA, particularly with conjugated double bonds, could be sensitive to heating below 100 °C, and that these conditions could favour the formation of CLA [67,69]. In contrast, other studies on cheese manufacture [12,72,73] found that different thermal treatments did not significantly alter total CLA content or the CLA isomer profile. Therefore, future studies will be designed to further investigate the effect of the processing and storage conditions of milk and dairy products on CLA content.

### 4.3. Volatile Compounds

Pasteurisation affects the volatile compound profile of cheese primarily by reducing milk’s indigenous microflora [74]. In our study, the milk used to manufacture FC was pasteurised, while RC samples included a starter culture. According to McSweeney [75], three sources of variability were likely involved in the volatile composition of our FC: enzymes from the rennet, indigenous milk enzymes (particularly plasmin and non-starter bacteria), and organisms that either survive pasteurisation of the milk or gain access to the pasteurised milk or curd during manufacture. In our analysis, the main families of compounds in FC were, in order of abundance: acids, alcohols, and ketones. A different sequence was observed in RC samples: alcohols, ketones, and acids. This finding agrees with Ortigosa et al. [76], who suggested that pasteurisation decreases the levels of alcohols and ketones. Juan et al. [77] also described a decrease of acids at the expense of alcohols and ketones in 15 and 60 d ewe cheese, but in this case, both kinds of cheese were pasteurized with the addition of a starter culture.

Branched-chain FAs, such as 3-methylbutanoic acid (present to a high extent in our FC samples), are derived from extensive proteolysis. Most straight-chain FAs that have between four and 20 carbon atoms, such as the hexanoic or octanoic acids detected especially in our RC samples, come from the lipolysis of triglycerides. In addition, a low proportion of FAs (e.g., acetic acid) can originate from the degradation of lactose or the oxidation of ketones, esters, or aldehydes [78].

Alcohols such as ethanol are derived primarily from the fermentation of multiple substrates such as lactose but also from amino acid metabolism and acetaldehyde reduction. Lactose fermentation can be carried out by a number of homofermentative bacteria, as well as by yeasts and leuconostocs [76]. Perhaps ethanol levels held steady in both kind of cheeses in our study, in FC because of the action of yeast (with less competition due to indigenous bacteria [79]), and in RC due to the action of the native microflora and the starter culture. Moreover, 3-methyl-1-butan-ol was also present in a high concentration in FC. Together with 3-methylbutanoic acid, this alcoholic compound is the product of the reduction/oxidation of an intermediate aldehyde (3-methyl-butanal) undetected in our samples; 3-methyl-butanal is derived from the deamination and decarboxylation of the amino acid leucine [78].

Butan-2-one-3-hydroxy (acetoin), the main volatile compound of FC, derives from lactose and citrate metabolism and likely plays an important role in the flavour of FC [80]. Acetoin is a common compound present in many other cheeses manufactured using ewe and goat milk [74,76,81,82]. *Lactococcus lactis* ssp. *lactis* and *Lactococcus lactis* ssp. *lactisbv*. *diacetylactis*, which were present in the starter culture used in the manufacture of RC, are primarily responsible for the production of diacetyl [74]. The diacetyl can be reduced to acetoin, which in turn can be reduced to butan-2,3-diol, then to butan-2-one, and finally to butan-2-ol [76]. These last two compounds accounted for 50% of the volatile fraction of RC.

Benzene-ethanol, a metabolic product of yeast that is derived from phenylalanine, was detected in both cheeses as the main aromatic compound; it is responsible for floral and rose flavour notes [83]. Regarding toluene, several researchers have suggested that the compound could originate from the degradation of carotene in milk [80].

Esters play a key role in cheese aroma because of their low sensory thresholds but also due to their high volatility at room temperatures [77]. High amounts of ethanol lead to the formation of many ethyl esters [81]. Ethyl acetate and ethyl hexanoate are produced by the esterification of alcohol and acetic and hexanoic acid by microorganisms or by chemical reaction. Ethyl acetate has been positively associated with sweet odour in Appenzeller cheese, whereas ethyl hexanoate is correlated with unripe apple overtones [78].

Regarding the miscellaneous minority compounds, we can emphasise the low quantity of aldehydes (transitory compounds due to their fast reduction or oxidation to alcohols or acids), lactones (formed by the cyclisation of γ- and δ-hydroxy acids), sulphur compounds (essentially originated from methionine degradation), and aliphatic hydrocarbons (secondary products of lipid autoxidation with a minor contribution to aroma) in the samples. Finally, we can also highlight the content in both cheese samples of farnesol, which is a terpene uncommonly detected in cheese that has been identified as a non-desirable aromatic compound due to its oil and boiled vegetable aromas [84].

## 5. Conclusions

This is the first assessment of quality and nutritional attributes of traditional dairy products from the Roja Mallorquina sheep breed, which are linked to geographic area of origin, highlighting specific traits, such as low percentage of fat and high content of antioxidant compounds. The variability in vitamins, phenolic compounds, antioxidant capacity, and FA was influenced by the cheese-making process (differences between the cheese and the original milk) and by the type of cheese-making technology (differences between the cheeses, mainly related to heating, the use of starter culture, and ripening). Fresh soft cheese, compared to the original milk and ripened cheese, was generally characterised by better nutritional value for human health according to the fat-soluble components—a favourable level of retention of retinol and α-tocopherol and a lower saturated FA percentage and lower atherogenic and thrombogenic indices. The fresh and ripened cheeses presented different volatile profiles derived from their distinct production methods.

Given the social and economic importance of sheep’s milk cheeses to small domestic producers in the Balearic Islands (Spain), this preliminary study can contribute to the recognition of the potential of the Roja Mallorquina native breed, add value to traditional products according to healthy nutritional criteria, and support the implementation of strategies to enhance commercialisation and obtain product labelling as “pasture-fed” or specific marks such as Protected Designation of Origin and Protected Geographical Indication. However, future studies are needed to further characterise the factors linked to geographical area of origin, including both natural (e.g., environment) and human (e.g., feeding management and production techniques) factors, and to investigate the relationship between the conditions of the cheese-making process and the nutritional value of cheese for human health.

## Figures and Tables

**Table 1:** Chemical composition of the main supplementary feeds during lactation.

Item	Chemical Composition (% Dry Matter, DM)				
	DM	Crude Protein	Crude Fibre	Ether Extract	Ash
Husks and broken cereals	88.8	17.4	8.50	2.24	6.57
Cereal mix (barley, 40%; oats, 40%; beans, 20%)	89.8	13.3	10.3	2.94	2.58
Oat hay	80.6	6.04	40.2	2.40	6.62
Barley straw	91.7	3.70	38.2	1.62	7.63
Oat silage	30.3	8.32	29.6	3.36	7.57

**Table 2:** Physicochemical composition of fresh and ripened Roja Mallorquina ewes’ cheese and corresponding original milk.

Item	Milk (*n* = 8)		Fresh Cheese (*n* = 8)		Ripened Cheese (*n* = 8)	
	Mean	SEM	Mean	SEM	Mean	SEM
Dry matter (DM) (%)	19.5	0.39	47.5	1.3	64.2	0.78
Lactose (%)	4.54	0.06				
Fat (%)	7.47	0.31	24.1	1.2	29.6	2.4
Protein (%)	6.57	0.18	14.6	0.34	25.6	0.33
Fat/DM (%)			50.7	1.5	46.2	4.7
Ash (%)			1.76	0.03	3.58	0.08
NaCl (%)			1.16	0.09	1.04	0.07
pH			6.54	0.03	5.27	0.03

**Table 3:** Fat-soluble vitamins, phenolic compounds, and total antioxidant capacity of fresh and ripened Roja Mallorquina ewes’ cheese and corresponding original milk.

Item ^1^	Milk (M, *n* = 8)		Fresh Cheese (FC, *n* = 8)		Ripened Cheese (RC, *n* = 8)		*P* ^2^		
	Mean	SEM	Mean	SEM	Mean	SEM	M vs. FC	M vs. RC	FC vs. RC
Retinol, μg/100 g DM	211.4	19.8	376.4	35.0	233.6	16.4	*	ns	**
Retinol, μg/g fat	5.56	0.45	7.89	0.77	5.45	0.58	†	ns	**
α-Tocopherol, μg/100 g DM	84.8	13.1	361.7	45.5	32.6	5.40	**	*	***
α-Tocopherol, μg/g fat	2.26	0.37	7.58	0.97	0.81	0.14	*	*	**
TPC, mg GA equivalents/100 g DM	18.7	0.54	6.16	0.55	54.5	4.44	***	**	***
TAC, μmol Trolox equivalents/g DM	22.4	6.98	100.0	12.7	102.5	11.2	*	**	ns

^1^ TPC, total phenolic compounds; GA, gallic acid; TAC, total antioxidant capacity; ^2^ Statistical probability for comparisons: ns, not significant (*p* > 0.05); *, *p* ≤ 0.05; **, *p* < 0.01; ***, *p* < 0.001; †, *p* ≤ 0.10.

**Table 4:** Fatty acid (FA) content (expressed as mg/g dry matter (DM)) of fresh and ripened Roja Mallorquina ewes’ cheese and corresponding original milk.

Item ^1^	Milk (M, *n* = 8)		Fresh Cheese (FC, *n* = 8)		Ripened Cheese (RC, *n* = 8)		*P* ^2^		
	Mean	SEM	Mean	SEM	Mean	SEM	M vs. FC	M vs. RC	FC vs. RC
C4:0	3.46	0.43	5.57	0.35	5.36	0.44	*	**	ns
C6:0	4.59	0.53	7.32	0.43	7.16	0.62	**	**	ns
C8:0	3.94	0.46	6.24	0.35	6.11	0.54	**	**	ns
C10:0	15.2	1.85	26.4	2.00	22.0	2.34	*	*	†
C11:0	0.29	0.03	0.45	0.05	0.38	0.03	†	ns	ns
C12:0	8.68	1.05	15.1	1.30	12.3	1.28	*	*	ns
C13:0	0.20	0.02	0.33	0.04	0.29	0.02	*	*	ns
C14:0	15.2	1.77	28.8	2.83	21.3	2.09	*	†	†
C14:1	0.62	0.07	1.25	0.15	0.87	0.08	**	*	*
C15:0	1.39	0.16	2.73	0.30	1.93	0.17	*	*	*
C15:1	0.11	0.01	0.77	0.20	0.13	0.01	**	ns	***
C16:0	44.4	5.68	81.1	10.9	49.6	4.92	*	ns	*
C16:1	2.10	0.28	3.80	0.45	2.46	0.21	*	ns	**
C17:0	0.93	0.11	1.72	0.21	1.28	0.17	*	ns	†
C17:1	0.56	0.10	1.24	0.18	0.61	0.06	*	ns	***
C18:0	21.1	2.32	37.6	5.82	19.8	2.15	†	ns	*
C18:1 n-9 *trans*	0.52	0.08	1.65	0.33	0.50	0.05	*	ns	**
C18:1 n-11 *trans* (VA)	1.98	0.22	3.47	0.51	1.79	0.17	*	ns	**
C18:1 n-9 *cis*	26.6	3.23	52.6	7.04	29.3	3.03	*	ns	*
C18:2 n-6 *trans*	0.51	0.07	1.09	0.11	0.71	0.09	*	ns	*
C18:2 n-6 *cis*	4.09	0.52	8.80	0.98	5.27	0.87	*	ns	*
γ-C18:3 n-6	0.11	0.02	0.31	0.05	0.31	0.04	*	**	ns
α-C18:3 n-3	1.08	0.14	2.60	0.38	1.24	0.15	*	ns	**
CLA *cis*-9, *trans*-11 (RA)	0.99	0.08	1.87	0.23	1.34	0.11	*	*	*
CLA *trans*-10, *cis*-12	0.06	0.01	0.25	0.03	0.09	0.01	*	ns	**
C20:0	0.35	0.04	0.17	0.03	0.12	0.01	**	**	ns
C20:1 n-9	0.14	0.01	0.14	0.01	0.11	0.02	ns	ns	ns
C20:2	0.11	0.02	0.21	0.02	0.12	0.01	*	ns	**
C20:3 n-3	0.06	0.01	0.23	0.05	0.29	0.03	**	***	ns
C20:3 n-6	0.07	0.01	0.15	0.00	0.12	0.01	**	*	†
C20:4 n-6 (ARA)	1.17	0.17	2.59	0.33	0.99	0.11	*	ns	**
C20:5 n-3 (EPA)	0.31	0.03	0.42	0.09	0.26	0.03	ns	ns	ns
C21:0	0.05	0.00	0.14	0.02	0.06	0.01	**	ns	***
C22:0	1.44	0.40	2.83	0.70	0.85	0.11	ns	ns	**
C22:1 n-9	0.10	0.01	0.16	0.01	0.13	0.02	†	†	ns
C22:2	0.06	0.01	0.08	0.01	0.03	0.00	ns	ns	*
C22:5 n-3 (DPA)	0.44	0.04	0.81	0.11	0.41	0.04	*	ns	**
C22:6 n-3 (DHA)	0.23	0.03	0.51	0.12	0.19	0.02	*	ns	**
C23:0	0.08	0.01	0.13	0.02	0.08	0.01	ns	ns	ns
C24:0	0.09	0.01	0.11	0.01	0.08	0.01	ns	ns	ns
C24:1	0.11	0.01	0.08	0.01	0.06	0.00	*	†	ns
SFA	121.5	14.1	216.9	24.9	148.7	14.7	*	ns	*
MUFA	32.9	3.91	65.2	8.75	36.0	3.51	*	ns	**
PUFA	9.32	1.03	19.9	2.37	11.4	1.31	*	ns	**
SCFA	27.2	3.10	45.6	2.99	40.6	3.86	**	*	ns
MCFA	74.5	9.16	137.3	16.4	91.2	8.97	*	ns	*
LCFA	62.0	7.15	119.1	16.7	64.3	6.74	*	ns	**
n-3	2.13	0.21	4.59	0.66	2.39	0.25	*	ns	**
n-6	5.96	0.75	12.9	1.43	7.41	0.99	*	ns	**
CLA total	1.05	0.09	2.12	0.26	1.44	0.12	*	*	*
n-6/n-3	2.76	0.13	2.91	0.14	3.08	0.17	ns	ns	ns
MUFA/SFA	0.27	0.01	0.30	0.01	0.24	0.00	ns	*	***
PUFA/SFA	0.08	0.00	0.09	0.00	0.08	0.00	*	ns	***
AI	2.71	0.08	2.52	0.08	3.11	0.02	ns	*	***
TI	3.08	0.09	2.78	0.05	3.11	0.02	ns	ns	**
HPI	0.37	0.01	0.40	0.01	0.32	0.00	ns	*	***

^1^ VA, vaccenic acid; RA, rumenic acid; ARA, arachidonic acid; EPA, eicosapentaenoic acid; DPA, docosapentaenoic acid; DHA, docosahexaenoic acid; SFA, saturated FAs; MUFA, monounsaturated FAs; PUFA, polyunsaturated FAs; SCFA, short-chain FAs; MCFA, medium-chain FAs; LCFA, long-chain FAs; CLA, conjugated linoleic acid; AI (atherogenic index; (C12:0 + 4 × 14:0 + C16:0)/(MUFA + PUFA)) and TI (thrombogenic index; (C14:0 + C16:0 + C18:0)/(0.5 × MUFA + 0.5 × n-6 + 3 × n-3 + n-3/n-6)) were computed using the procedure proposed by Ulbricht and Southgate [37]; health-promoting index (HPI) was the inverse of AI, [38]; ^2^ Statistical probability for comparisons: ns, not significant (*p* > 0.05); *, *p* ≤ 0.05; **, *p* < 0.01; ***, *p* < 0.001; †, *p* ≤ 0.10.

**Table 5:** Fatty acid (FA) profile (expressed as % of total FA) of fresh and ripened Roja Mallorquina ewes’ cheese and corresponding original milk.

Item ^1^	Milk (M)		Fresh Cheese (FC)		Ripened Cheese (RC)		*P* ^2^		
	Mean	SEM	Mean	SEM	Mean	SEM	M vs. FC	M vs. RC	FC vs. RC
C4:0-C10:0	14.7	0.85	13.7	0.68	18.3	0.22	ns	**	**
C12:0	5.21	0.12	5.03	0.19	6.20	0.09	*	**	***
C14:0	9.46	0.05	9.83	0.30	11.1	0.11	ns	**	***
C16:0	27.9	0.68	27.4	0.44	26.3	0.06	ns	†	ns
C18:0	13.5	0.21	12.7	0.41	10.6	0.22	†	***	**
C18:1 n-9 *cis*	16.9	0.55	18.00	0.21	15.6	0.08	*	ns	***
CLA *cis*-9, *trans*-11 (RA)	0.54	0.04	0.53	0.03	0.61	0.04	ns	*	ns
Others	11.8	0.23	12.8	0.30	11.3	0.05	ns	ns	**
SFA	73.3	0.61	71.1	0.35	74.9	0.09	*	ns	***
MUFA	20.9	0.53	22.2	0.35	19.2	0.05	*	†	***
PUFA	5.78	0.18	6.67	0.08	5.87	0.14	*	ns	**
n-3	1.36	0.07	1.54	0.06	1.26	0.03	†	*	**
n-6	3.74	0.13	4.44	0.05	3.88	0.17	*	ns	**

^1^ SFA, saturated FAs; MUFA, monounsaturated FAs; PUFA, polyunsaturated FAs; SCFA, short-chain FAs; MCFA, medium-chain FAs; LCFA, long-chain FAs; CLA, conjugated linoleic acid; ^2^ Statistical probability for comparisons: ns, not significant (*p* > 0.05); *, *p* ≤ 0.05; **, *p* < 0.01; ***, *p* < 0.001; †, *p* ≤ 0.10.

## Data Availability

The data presented in this study are available on request from the corresponding autor.

## References

[1] Morales-Jerrett E., Mancilla-Leytón J.M., Delgado-Pertíñez M., Mena Y. (2020). The contribution of traditional meat goat farming systems to human wellbeing and its importance for the sustainability of this livestock subsector. Sustainability.

[2] ARCA Ovella Roja Mallorquina. https://www.mapa.gob.es/es/ganaderia/temas/zootecnia/razas-ganaderas/razas/catalogo-razas/ovino/roja-mallorquina/default.aspx..

[3] Vinyoles i Vidal T.M. (1991). Notes sobre el formatge de Mallorca. Bolletí Soc. Arqueol. Lul·Liana.

[4] Artaud-Wild S.M., Connor S., Sexton G., Connor W.E. (1993). Differences in coronary mortality can be explained by differences in cholesterol and saturated fat intakes in 40 countries but not in France and Finland. A paradox. Circulation.

[5] Lordan R., Tsoupras A., Mitra B., Zabetakis I. (2018). Dairy fats and cardiovascular disease: Do we really need to be concerned?. Foods.

[6] McGrath J., Duval S.M., Tamassia L.F.M., Kindermann M., Stemmler R.T., de Gouvea V.N., Acedo T.S., Immig I., Williams S.N., Celi P. (2018). Nutritional strategies in ruminants: A lifetime approach. Res. Vet. Sci..

[7] Khan I.T., Nadeem M., Imran M., Ullah R., Ajmal M., Jaspal M.H. (2019). Antioxidant properties of Milk and dairy products: A comprehensive review of the current knowledge. Lipids Health Dis..

[8] Elgersma A., Tamminga S., Ellen G. (2006). Modifying milk composition through forage. Anim. Feed Sci. Technol..

[9] Capuano E., van der Veer G., Boerrigter-Eenling R., Elgersma A., Rademaker J., Sterian A., van Ruth S.M. (2014). Verification of fresh grass feeding, pasture grazing and organic farming by cows farm milk fatty acid profile. Food Chem..

[10] Rubino R. (2014). A special section on Latte Nobile: An evolving model. J. Nutrit. Ecol. Food Res..

[11] Méndez C. (2018). La leche fresca de Lidl, la primera que cuenta con certificado de pastoreo y de bienestar animal. Aral.

[12] Gnädig S., Chamba J.F., Perreard E., Chappaz S., Chardigny J.M., Rickert R., Steinhart H., Sébédio J.L. (2004). Influence of manufacturing conditions on the conjugated linoleic acid content and the isomer composition in ripened French Emmental cheese. J. Dairy Res..

[13] Valdivielso I., Bustamante M.Á., Buccioni A., Franci O., Ruíz de Gordoa J.C., de Renobales M., Barron L.J.R. (2015). Commercial sheep flocks–fatty acid and fat-soluble antioxidant composition of milk and cheese related to changes in feeding management throughout lactation. J. Dairy Res..

[14] Bodkowski R., Czyż K., Kupczyński R., Patkowska-Sokoła B., Nowakowski P., Wiliczkiewicz A. (2016). Lipid complex effect on fatty acid profile and chemical composition of cow milk and cheese. J. Dairy Sci..

[15] Laskaridis K., Serafeimidou A., Zlatanos S., Gylou E., Kontorepanidou E., Sagredos A. (2013). Changes in fatty acid profile of feta cheese including conjugated linoleic acid. J. Sci. Food Agric..

[16] Bocquel D., Marquis R., Dromard M.P., Salamin A., Rey-Siggen J., Héritier J., Kosinska-Cagnazzo A., Andlauer W. (2016). Effect of flaxseed supplementation of dairy cows’ forage on physicochemical characteristic of milk and Raclette cheese. Int. J. Dairy Technol..

[17] Schiavon S., Cesaro G., Cecchinato A., Cipolat-Gotet C., Tagliapietra F., Bittante G. (2016). The influence of dietary nitrogen reduction and conjugated linoleic acid supply to dairy cows on fatty acids in milk and their transfer to ripened cheese. J. Dairy Sci..

[18] Lucas A., Rock E., Chamba J.F., Verdier-Metz I., Brachet P., Coulon J.B. (2006). Respective effects of milk composition and the cheese-making process on cheese compositional variability in components of nutritional interest. Lait.

[19] Cifre J., Rigo A., Gulías J., Rallo J., Joy M., Joy S., Mus M., Sánchez F., Ramón J., Ruiz M., Conselleria d´Agricultura i Pesca (2007). Caracterizaciò de les pastures de les Illes Balears. Quaderns dÍnvestigación 7.

[20] Horwitz W., Latimer G., AOAC (2005). Association of Official Analytical Chemist. Official Methods of Analysis.

[21] Delgado-Pertíñez M., Alcalde M.J., Guzmán-Guerrero J.L., Castel J.M., Mena Y., Caravaca F. (2003). Effect of hygiene-sanitary management on goat milk quality in semi-extensive systems in Spain. Small Rumin. Res..

[22] ISO/IDF (2008). Cheese—Determination of Fat Content—Van Gulik Method.

[23] Cunniff P., AOAC (1999). Association of Official Analytical Chemists. Official Methods of Analysis.

[24] Helrich K., AOAC (1990). Association of Official Analytical Chemists. Official Methods of Analysis.

[25] De la Haba Ruiz M., Ruiz Pérez-Cacho P., Dios Palomares R., Galan-Soldevilla H. (2016). Classification of artisanal Andalusian cheeses on physicochemical parameters applying multivariate statistical techniques. Dairy Sci. Technol..

[26] Guzmán J.L., Pérez-Écija A., Zarazaga L.A., Martín-García A.I., Horcada A., Delgado-Pertíñez M. (2020). Using dried orange pulp in the diet of dairy goats: Effects on milk yield and composition and blood parameters of dams and growth performance and carcass quality of kids. Animal.

[27] Sukhija P.S., Palmquist D.L. (1988). Rapid method for determination of total fatty acid content and composition of feedstuffs and feces. J. Agri. Food Chem..

[28] Juárez M., Polvillo O., Contò M., Ficco A., Ballico S., Failla S. (2008). Comparison of four extraction/methylation analytical methods to measure fatty acid composition by gas chromatography in meat. J. Chromatogr. A.

[29] Herrero-Barbudo M.C., Granado-Lorencio F., Blanco-Navarro I., Olmedilla-Alonso B. (2005). Retinol, α-and γ-tocopherol and carotenoids in natural and vitamin A- and E-fortified dairy products commercialized in Spain. Int. Dairy J..

[30] Chauveau-Duriot B., Doreau M., Nozière P., Graulet B. (2010). Simultaneous quantification of carotenoids, retinol, and tocopherols in forages, bovine plasma, and milk: Validation of a novel UPLC method. Anal. Bioanal. Chem..

[31] Gutiérrez-Peña R., Fernández-Cabanás V.M., Mena Y., Delgado-Pertíñez M. (2018). Fatty acid profile and vitamins A and E contents of milk in goat farms under Mediterranean wood pastures as affected by grazing conditions and seasons. J. Food Compost. Anal..

[32] Pellegrini N., Ke R., Yang M., Rice-Evans C. (1999). Screening of dietary carotenoids and carotenoid-rich fruit extracts for antioxidant activities applying 2,2 0-azinobis(3-ethylenebenzothiazoline-6-sulfonic acid) radical catión decolourisation assay. Meth. Enzymol..

[33] Delgado-Pertíñez M., Gutiérrez-Peña R., Mena Y., Fernández-Cabanás V.M., Laberye D. (2013). Milk production, fatty acid composition and vitamin E content of Payoya goats according to grazing level in summer on Mediterranean shrublands. Small Rumin. Res..

[34] Revilla I., González-Martín M.I., Vivar-Quintana A.M., Blanco-López M.A., Lobos-Ortega I.A., Hernández-Hierro J.M. (2016). Antioxidant capacity of different cheeses: Affecting factors and prediction by near infrared spectroscopy. J. Dairy Sci..

[35] Vázquez C.V., Rojas M.G., Ramirez C.A., Chávez-Servin J.L., García-Gasca T., Ferriz-Martínez R.A., Garcia O.P., Rosado J.L., López-Sabater C.M., Castellote A.I. (2015). Total phenolic compounds in milk from different species. Design of an extraction technique for quantification using the Folin-Ciocalteu method. Food Chem..

[36] Guzmán J.L., Delgado Pertíñez M., Galán Soldevilla H., Ruiz Pérez-Cacho P., Polvillo Polo O., Zarazaga L.Á., Avilés Ramírez C. (2020). Effect of citrus by-product on physicochemical parameters, sensory analysis and volatile composition of different kinds of cheese from raw goat milk. Foods.

[37] Ulbricht T.L.V., Southgate D.A.T. (1991). Coronary heart disease: Seven dietary factors. Lancet.

[38] Chen S., Bobe G., Zimmerman S., Hammond E.G., Luhman C.M., Boylston T.D., Freeman A.E., Beitz D.C. (2004). Physical and sensory properties of dairy products from cows with various milk fatty acid compositions. J. Agric. Food Chem..

[39] Jaramillo D.P., Zamora A., Guamisa B., Rodríguez M., Trujillo A.J. (2008). Cheesemaking aptitude of two Spanish dairy ewe breeds: Changes during lactation and relationship between physico-chemical and technological properties. Small Rumin. Res..

[40] Estrada O., Ariño A., Juan T. (2019). Salt Distribution in Raw Sheep Milk Cheese during Ripening and the Effect on Proteolysis and Lipolysis. Foods.

[41] Hernández I., Barrón L.J.R., Virto M., Pérez-Elortondo F.J., Flanagan C., Rozas U., Nájera A.I., Albisu M., Vicente M.S., de Renobales M. (2009). Lipolysis, proteolysis and sensory properties of ewe’s raw milk cheese (Idiazabal) made with lipase addition. Food Chem..

[42] Irigoyen A., Izco J.M., Ibáñez F.C., Torre P. (2002). Influence of calf or lamb rennet on the physicochemical, proteolytic, and sensory characteristics of an ewes milk cheese. Int. Dairy J..

[43] Cabezas L., Sánchez I., Poveda J.M., Seseña S., Palop M.L.L. (2007). Comparison of microflora, chemical and sensory characteristics of artisanal Manchego cheeses from two dairies. Food Control.

[44] San Juan E., Millan R., Saavedra P., Carmona M.A., Gómez R., Fernandez-Salguero J. (2002). Influence of animal and vegetable rennet on the physicochemical characteristics of Los Pedroches cheese during ripening. Food Chem..

[45] Valdivielso I., Bustamante M.A., Aldezabal A., Amores G., Virto M., Ruíz de Gordoa J.C., de Renobales M., Barron L.J.R. (2016). Case study of a commercial sheep flock under extensive mountain grazing: Pasture derived lipid compounds in milk and cheese. Food Chem..

[46] De Man J.M. (1981). Light-induced destruction of vitamin A in milk. J. Dairy Sci..

[47] Poiffait A., Lietaer E., Le Pavec P., Adrian J. (1992). Interrelations physico-chimiques et nutritionnelles entre la caséine et les vitamines liposolubles. Ind. Aliment. Agric..

[48] Bansal V., Mishra S.K. (2020). Reduced-sodium cheeses: Implications of reducing sodium chloride on cheese quality and safety. Compr. Rev. Food Sci. Food Saf..

[49] do Oriente S.F., Barreto F., Tomaszewski C.A., Barnet L.S., Souza N.C., Oliveira H.M., de Bittencourt M.A. (2020). Retention of vitamin A after goat milk processing into cheese: A nutritional strategy. J. Food Sci. Technol..

[50] Poulopoulou I., Zoidis E., Massouras T., Hadjigeorgiou I. (2012). Terpenes transfer to milk and cheese after oral administration to sheep fed indoors. J. Anim. Physiol. Anim. Nutr..

[51] Abd El-Gawad M.A.M., Ahmed N.S. (2011). Cheese yield as affected by some parameters. Review. Acta Sci. Pol. Technol. Aliment..

[52] Chávez-Servín J.L., Andrade-Montemayor H.M., Velázquez Vázquez C., Aguilera Barreyro A., García-Gasca T., Ferríz Martínez R.A., Olvera Ramírez A.M., de la Torre-Carbot K. (2018). Effects of feeding system, heat treatment and season on phenolic compounds and antioxidant capacity in goat milk, whey and cheese. Small Rumin. Res..

[53] Nagy S., Rouseff R., Lee H. (1989). Thermally degraded flavors in citrus juice products. ACS Symp. Ser..

[54] Chen Y., Yu L.J., Rupasinghe H.P. (2013). Effect of thermal and non-thermal pasteurization on the microbial inactivation and phenolic degradation in fruit juice: A mini-review. J. Sci. Food Agric..

[55] Di T.A., Bonanno A., Cecchini S., Giorgio D., Di G.A., Claps S. (2015). Effects of Sulla forage (*Sulla coronarium* L.) on the oxidative status and milk polyphenol content in goats. J. Dairy Sci..

[56] Zulueta A., Maurizi A., Frígola A., Esteve M.J., Coli R., Burini G. (2009). Antioxidant capacity of cow milk, whey and deproteinized milk. Int. Dairy J..

[57] Virto M., Bustamante M., de Gordoa J.C.R., Amores G., Fernández-Caballero P.N., Mandaluniz N., Arranz J., Nájera A.I., Albisu M., Pérez-Elortondo F.J. (2012). Interannual and geographical reproducibility of the nutritional quality of milk fat from commercial grazing flocks. J. Dairy Res..

[58] Abilleira E., Collomb M., Schlichtherle-Cerny H., Virto M., de Renobales M., Barron L.J.R. (2009). Winter/Spring changes in fatty acid composition of farmhouse Idiazabal cheese due to different flock management systems. J. Agric. Food Chem..

[59] Bergamaschi M., Bittante G. (2017). Detailed fatty acid profile of milk, cheese, ricotta and by products, from cows grazing summer highland pastures. J. Dairy Res..

[60] Pérez M.D., Calvo M. (1995). Interaction of β-lactoglobulin with retinol and fatty acids and its role as a possible biological function for this protein: A review. J. Dairy Sci..

[61] Martino C., Ianni A., Grotta L., Pomilio F., Martino G. (2019). Influence of Zinc Feeding on Nutritional Quality, Oxidative Stability and Volatile Profile of Fresh and Ripened Ewes’ Milk Cheese. Foods.

[62] Molimard P., Spinnler H.E. (1996). Review: Compounds involved in the flavour of surface mould-ripened cheeses: Origins and properties. J. Dairy Sci..

[63] Gil A., Martínez de Victoria E., Olza J. (2015). Indicators for the evaluation of diet quality. Nutr. Hosp..

[64] Molendi-Coste O., Legry V., Leclercq I.A. (2011). Why and how meet n-3 PUFA dietary recommendations? A review. Gastroenterol. Res. Pract..

[65] MacRae J., O’Reilly L., Morgan P. (2005). Desirable characteristics of animal products from a human health perspective. Livest. Prod. Sci..

[66] Palmquist D.L., Lock A.L., Shingfield K.J., Bauman D.E. (2005). Biosynthesis of conjugated linoleic acid in ruminants and humans. Adv. Food Nutr. Res..

[67] Ha Y.L., Grimm N.K., Pariza M.W. (1989). Newly recognized anticarcinogenic fatty acids: Identification and quantification in natural and processed cheeses. J. Agric. Food Chem..

[68] Shantha N.C., Decker E.A. (1993). Conjugated linoleic acid concentrations in processed cheese containing hydrogen donors, iron and dairy based additives. Food Chem..

[69] Shantha N.C., Decker E.A., Ustunol Z. (1992). Conjugated linoleic acid concentration in processed cheese. J. Am. Oil Chem. Soc..

[70] Buccioni A., Rapaccini S., Antongiovanni M., Minieri S., Conte G., Mele M. (2010). Conjugated linoleic acid and C18:1 isomers content in milk fat of sheep and their transfer to Pecorino Toscano cheese. Int. Dairy J..

[71] Avilez J.P., Wladimir M., Delgado-Pertiñez M. (2016). Conjugated linoleic acid of dairy foods is affected by cow’s feeding system and processing of milk. Sci. Agric..

[72] Luna P., De la Fuente M.A., Juárez M. (2005). Conjugated linoleic acid in processed cheeses during the manufacturing stages. J. Agric. Food Chem..

[73] Luna P., Juárez M., De la Fuente M.A. (2007). Conjugated linoleic acid content and isomer distribution during ripening in three varieties of cheeses protected with designation of origin. Food Chem..

[74] Buchin S., Delague V., Duboz G., Berdague J.L., Beuvier E., Pochet S., Grappin R. (1998). Influence of pasteurization and fat composition of milk on the volatile compounds and flavor characteristics of a semi-hard cheese. J. Dairy Sci..

[75] McSweeney P., Sousa-Gallagher M.J. (2000). Biochemical pathways for the production of flavour compounds in cheeses during ripening: A review. Lait.

[76] Ortigosa M., Torre P., Izco J.M. (2001). Effect of pasteurization of ewe’s milk and use of a native starter culture on the volatile components and sensory characteristics of Roncal cheese. J. Dairy Sci..

[77] Juan B., Barron L.J.R., Ferragut V., Trujillo A.J. (2007). Effects of high pressure treatment on volatile profile during ripening of ewe milk cheese. J. Dairy Sci..

[78] Curioni P.M.G., Bosset J.O. (2002). Key odorants in various cheese types as determined by gas chromatography-olfactometry. Int. Dairy J..

[79] Olarte C. (1999). Caracterización del Queso de Cameros. Evolución de Parámetros Fisicoquímicos y Microbiológicos Durante su Maduración. Ph.D. Thesis.

[80] Atasoy A.F., Hayaloglu A.A., Kırmacı H., Levent O., Türkoğlu H. (2013). Effects of partial substitution of caprine for ovine milk on the volatile compounds of fresh and mature Urfa cheeses. Small Rumin. Res..

[81] Karali F., Georgala A., Massouras T., Kaminarides S. (2013). Volatile compounds and lipolysis levels of Kopanisti, a traditional Greek rawmilk cheese. J. Sci. Food Agric..

[82] Quintanilla P., Hettinga K.A., Beltrán M.C., Escriche I., Molina M.P. (2020). Short communication: Volatile profile of matured Tronchón cheese affected by oxytetracycline in raw goat milk. J. Dairy Sci..

[83] Güler Z. (2014). Profiles of organic acid and volatile compounds in acid-type cheeses containing herbs and spices (Surk cheese). Int. J. Food Prop..

[84] Risner D., Tomasino E., Hughes P., Meunier-Goddik L. (2019). Volatile aroma composition of distillates produced from fermented sweet and acid whey. J. Dairy Sci..

